# Sun Exposure and Behaviours in Saudi Arabia: A National Study of over Eleven Thousand Participants Utilising the Arabic Sun Exposure and Behaviour Inventory

**DOI:** 10.3390/healthcare13233078

**Published:** 2025-11-26

**Authors:** Abdullah Aleisa, Nasser M. AbuDujain, Qais A. Almuhaideb, Yazeed A. Alrodiman, Hadeel F. AlMajid, Turki N. AboSarhad, Omnia A. Alali, Anas M. Abudasir, Dana Alkhudair, Ibtihal Alshammari, Inge J. Veldhuizen, Khalid F. Alsadhan, Abdullah A. Alrasheed, Saad M. Alsaad, Turky H. Almigbal

**Affiliations:** 1 Department of Dermatology, College of Medicine, King Saud University, Riyadh 11472, Saudi Arabia; 2 Department of Family and Community Medicine, College of Medicine, King Saud University, Riyadh 11495, Saudi Arabia; dr.khalidfm@gmail.com (K.F.A.); drabdullah99@hotmail.com (A.A.A.); salsaad@ksu.edu.sa (S.M.A.); talmigbal@ksu.edu.sa (T.H.A.); 3 Division of Dermatology, McGill University, Montréal, QC H4A 3J1, Canada; qaissalmuhaideb@gmail.com; 4 College of Medicine, Alfaisal University, Riyadh 11533, Saudi Arabia; yalrodiman@alfaisal.edu (Y.A.A.); hadeelfalmajid@gmail.com (H.F.A.); 5 College of Medicine, King Khalid University, Abha 61421, Saudi Arabia; turki.asa3@gmail.com (T.N.A.); anas.dasir.med@gmail.com (A.M.A.); 6 College of Medicine, University of Tabuk, Tabuk 71491, Saudi Arabia; omniaalali16@gmail.com; 7 College of Medicine, Imam Abdulrahman Bin Faisal University, Dammam 31441, Saudi Arabia; alkhudairdana@gmail.com; 8 College of Medicine, Hail University, Hail 55476, Saudi Arabia; ebtehal.shammar@gmail.com; 9 Department of Plastic and Reconstructive Surgery, Catharina Hospital, 5623 EJ Eindhoven, The Netherlands; ingejveldhuizen@gmail.com; 10 Prince Faisal Bin Bandar Chair for Geriatric Research, College of Medicine, King Saud University, Riyadh 11451, Saudi Arabia

**Keywords:** sun exposure, behaviours, UV protection, skin cancer, Saudi Arabia

## Abstract

**Background**: Excessive sun exposure is a major modifiable risk factor for skin cancer, with ultraviolet (UV) radiation identified as a key contributor. Saudi Arabia’s high UV index and hot climate increase the risk of photo-induced skin damage among residents. Although awareness of sun protection is growing, inconsistent use of preventive measures persists, often due to misconceptions and limited knowledge. This study aimed to assess patterns of current and prior sun exposure across demographic groups in Saudi Arabia using the validated Arabic version of the Sun Exposure and Behaviour Inventory (Ar-SEBI). **Methods**: An analytical cross-sectional study was conducted between August 2024 and January 2025 across all five Saudi regions using non-probability quota sampling to ensure geographic representation. Sun-exposure practices and protective behaviours were measured using the Ar-SEBI. Data were collected via an online questionnaire and analysed using IBM SPSS Statistics 26. Normality was assessed via Shapiro–Wilk and Levene’s tests. Depending on the distribution, data were analysed using the Mann–Whitney U test, the Kruskal–Wallis H test, unpaired *t*-tests, or ANOVA. A *p*-value < 0.05 was considered statistically significant. **Results**: A total of 11,491 adults participated. Significant demographic and regional differences in sun-related behaviours were observed. Overall, 72.7% of participants were unaware of the SPF level of their sunscreen. Males and individuals under 30 years of age had significantly higher sun exposure scores than females and older adults, respectively (*p* < 0.001). Residents of the Southern region had the highest exposure and behaviour scores (*p* < 0.001), despite also reporting higher engagement in some protective measures. Additionally, sun exposure scores increased progressively with darker Fitzpatrick skin types (IV–VI) (*p* < 0.001), suggesting a common belief that darker skin confers adequate protection. **Conclusions**: This study reveals notable disparities in sun-protection practices across Saudi Arabia. Younger adults, males, and individuals with darker skin types reported greater sun exposure and indicators of lower protective awareness. These findings support the need for targeted, culturally tailored public health campaigns and policy measures to enhance sun safety and reduce long-term dermatological risks.

## 1. Introduction

Excessive ultraviolet (UV) radiation is widely recognised as the primary preventable risk factor for skin cancers, including melanoma and non-melanoma types [1,2]. High UV radiation exposure directly damages DNA in epidermal cells, significantly contributing to skin carcinogenesis [3,4]. Saudi Arabia, characterised by its hot, arid climate and consistently high UV indices throughout the year, places its residents at increased risk for UV-induced skin damage and related malignancies [5,6]. Despite these recognised risks, adherence to sun-protective behaviours, such as sunscreen application, seeking shade, and wearing protective clothing, remains inconsistent among the Saudi population, influenced by misconceptions about sun safety and limited awareness [7].

Recent studies have highlighted persistent gaps in public knowledge regarding adequate sun protection, including uncertainty about the appropriate use of sunscreen, SPF ratings, and the optimal use of protective clothing, further complicating preventive efforts [8,9]. Additionally, sociocultural attitudes toward tanning and aesthetic perceptions associated with sun exposure may reduce the motivation to adopt effective protective behaviours, leading to higher cumulative UV exposure in the population [10]. Moreover, rapid urbanisation and changing lifestyle patterns have led to an increase in outdoor leisure activities among young adults, necessitating updated insights into sun-related behaviours to inform culturally tailored public health interventions [11].

Recent reports suggest that skin cancer, once thought to be relatively uncommon in Saudi Arabia compared with Western countries, has been increasing gradually over the past decade and now deserves closer public health attention. Data from regional and national studies between 2011 and 2022 show a steady rise in both melanoma and non-melanoma types. In the Aseer region, Algarni et al. (2023) [12] reviewed 560 confirmed cases and found that squamous cell carcinoma accounted for about 41% and basal cell carcinoma for 26%, mostly affecting the head and neck (55%) and occurring more often in men and older adults. On a national scale, AlOtaibi et al. (2023) [13] reported a similar pattern, with 37% squamous cell carcinoma, 31% basal cell carcinoma, and only 4% melanoma. A broader review by Aljabri et al. (2025) [14] estimated that skin cancer represents roughly 2% of all new cancer cases in Saudi Arabia, emphasising that the burden, though still lower than in Western populations, is increasing. Despite high ultraviolet exposure, protective cultural clothing may reduce risk to some extent, yet emerging lifestyle changes, increased outdoor recreation, and evolving fashion trends have introduced new patterns of sun exposure. Furthermore, awareness and knowledge of skin cancer prevention among the Saudi population remain limited, as many individuals underestimate the need for sunscreen or regular dermatologic screening [15].

To bridge these knowledge gaps, this study utilises the Arabic Sun Exposure and Behaviour Inventory (Ar-SEBI) to objectively evaluate sun-exposure practices and behaviours among a diverse Saudi cohort. The goal is to generate critical data that can inform targeted, culturally sensitive public health interventions, effectively balancing UV risk mitigation.

## 2. Methodology

### 2.1. Study Design and Setting

An analytical cross-sectional investigation was conducted from August 2024 to January 2025 in multiple regions throughout Saudi Arabia to evaluate sun-exposure behaviours and protective practices. Target regions were selected to reflect the country’s geographic diversity and varying climatic conditions, thereby allowing a more comprehensive assessment of ultraviolet (UV) exposure patterns.

### 2.2. Participants and Sampling

A non-probability quota sampling technique was implemented. The strata were categorised based on the region of living (i.e., central, eastern, western, northern, and southern). The sample size of this study was calculated based on the available national statistics of citizens in Saudi Arabia, as reported by the General Authority for Statistics in mid-2024 [16]. The total population was reported to be 35,300,280 citizens. Using the formula of known population with a 99% confidence interval and a margin of error of 5%, the minimum sample size is determined to be 664. To fulfil representative data, a minimum of 664 participants per region were targeted (a total of 3320 participants from all regions).

Inclusion criteria were tied to living in Saudi Arabia, being at least 18 years of age, and being fluent in Arabic. Individuals of any nationality were qualified for inclusion if they resided in Saudi Arabia during the data collection window, while Saudi citizens living abroad at the time were excluded. A multi-channel recruitment strategy leveraged social media platforms (e.g., WhatsApp, Telegram) and other community outreach initiatives to ensure broad demographic coverage across different regions.

### 2.3. Instrument: The Sun Exposure and Behaviour Inventory (SEBI)

Sun exposure and protective behaviours were evaluated using the Sun Exposure and Behaviour Inventory (SEBI), a validated self-report measure developed by Jennings et al. [17]. The SEBI comprises three core domains:

1. Current Sun Exposure—Frequency and duration of outdoor activities (e.g., work, sports) alongside the typical times of day individuals are in direct sunlight.
2. Current Sun Behaviour—Regularity of sunscreen application (including SPF and UVA/UVB considerations), use of protective clothing (e.g., hats, long sleeves), and shade-seeking.
3. Prior Sun Exposure—Historical accounts of prolonged sun exposure or severe sunburn episodes, emphasising cumulative UV risk.

Each SEBI domain is scored according to the original guidelines, resulting in composite values that reflect either higher sun exposure or less protective behaviour, depending on the domain [18]. Previous studies have employed the SEBI in diverse populations, establishing its reliability for characterising sun-related risk profiles [17]. We used the Arabic version of SEBI (Ar-SEBI), which has been validated and cross-culturally adapted, exhibiting good metrics [19]. Each SEBI domain (current sun exposure, current sun behaviour, and prior sun exposure) was scored according to the original SEBI guidelines described by Jennings et al. [17]. Each item contributes to a composite domain score, where higher values represent either greater sun exposure or lower adherence to protective practices, depending on the domain. Specifically, the possible range for *current sun behaviour* and *current sun exposure* scores is 0–200, and for *prior sun exposure* is 0–100. The total overall SEBI score, representing the combined sum of all domains, therefore ranges from 0 to 300, with higher total scores indicating higher cumulative sun exposure and less protective behaviour. The scoring system and scale weighting were identical to those used in prior international applications of the SEBI, ensuring full comparability of the results across populations.

In this scoring system, higher SEBI scores correspond to greater sun exposure or poorer adherence to protective behaviours, while lower scores indicate better adherence to sun protection practices and less cumulative UV exposure. This inverse relationship was maintained across all domains to ensure consistency with the original SEBI interpretation. High domain scores in *current sun exposure* specifically represent greater frequency or duration of time spent outdoors, while high *prior sun exposure* scores reflect a history of prolonged or frequent UV exposure. Thus, higher values consistently correspond to greater exposure across all SEBI components.

In addition, participants were asked to self-identify their skin type using the standard Fitzpatrick Skin Phototype Classification (Types I–VI), based on descriptions provided within the questionnaire regarding their natural skin colour, tendency to burn, and ability to tan after sun exposure.

To ensure contextual relevance, items within the SEBI related to clothing (SEBI 5 and SEBI 6) were interpreted in light of traditional Saudi attire. Female participants wearing the abaya and headscarf (and in some cases, facial covering) and male participants wearing the thoub and ghutra were instructed to respond according to their usual outdoor dress practices. In this context, “long-sleeved shirts” corresponded to traditional garments providing full arm and leg coverage, while “wearing a hat” was clarified as any additional form of sun-shielding headwear beyond customary cultural coverings. This clarification was included in the questionnaire introduction to ensure accurate cultural adaptation of the original SEBI items.

For SEBI items 14–16, which required participants to compare their lifetime sun exposure to that of “other people of the same age and sex,” additional clarification was provided to enhance response consistency. Participants were instructed to consider individuals within their own community and region (e.g., peers, friends, and family members living under similar climatic conditions) as the reference group. This guidance was included in the Arabic version of the SEBI (Ar-SEBI) to ensure culturally and contextually meaningful comparisons across respondents.

### 2.4. Ethical Approval and Procedures

All study procedures were approved by the King Saud University Institutional Review Board (No. E-24-8637, Ref. No. 24/1178/IRB) and conducted under the Declaration of Helsinki. Participants received detailed information regarding the study’s objectives and data confidentiality procedures and provided electronic informed consent.

### 2.5. Data Collection and Management

Data were collected using a self-administered, structured questionnaire developed by the research team. The questionnaire was distributed through an online form, and responses were collected electronically. The online format allowed broad geographic reach and participant convenience while maintaining anonymity.

After data cleaning, the final analytic sample consisted of 11,491 valid responses. Minor variations in subgroup totals within Table 1 reflect missing responses for certain demographic items (in table caption). The missing data were minimal and did not affect percentage calculations or overall analyses.

### 2.6. Statistical Analysis

SEBI scores were compared for the demographic and clinical variables. Non-normally distributed data, measured with the Shapiro–Wilk test and Levene’s test, were tested with the Mann–Whitney U or Kruskal–Wallis H test; otherwise, the unpaired *t*-test or ANOVA was applied. A *p* < 0.05 was considered statistically significant. Data management and analysis were performed using IBM SPSS Statistics 26.0 (IBM, Armonk, NY, USA). The total (combined) SEBI score was calculated as the sum of the three domain scores (current sun exposure, current sun behaviour, and prior sun exposure), representing an overall measure of cumulative exposure.

## 3. Results

A total of 11,491 participants were included in the study, with a response rate of 72.7% (out of 15,800 who opened the survey). The sample had a notable female predominance (71.1%) and a mean age of 29.6 ± 10.9 years. Over half (56.9%, *n* = 6508) were younger than 30 years old. Most participants held at least a bachelor’s degree (69.7%) and reported a monthly income of less than 5000 SAR (60.6%). Geographically, participants were predominantly from the Central (26.8%) and Eastern (22.4%) regions, followed by the Southern (18.7%), Western (18.4%), and Northern (13.6%) regions. Fitzpatrick skin types II (34.2%) and III (40.5%) were the most common. A small proportion reported a prior diagnosis of skin cancer (0.3%, *n* = 32) or use of immunosuppressive therapy (3.6%) (Table 1).

Participants generally showed modest adherence to protective behaviours (Table 2). Only 9.9% reported consistently applying sunscreen during extended outdoor activities, and a significant portion (72.7%) either used sunscreens with an SPF below 15 or were unsure of the SPF. UVA/UVB protection was confirmed by 28.1%. More than half of the participants (56.1%) habitually wore long-sleeved clothing outdoors, while other protective behaviours, such as seeking shade (16.8%) and wearing wide-brimmed hats (43.2%), were less frequently adopted.

Descriptive statistics indicated mean scores of 87.68 ± 46.17 for current sun behaviour, 47.87 ± 43.95 for current sun exposure, and 29.60 ± 27.50 for prior sun exposure, with higher scores indicating riskier practices or increased exposure. The total overall score was 165.20 ± 84.50 across all 11,491 participants. Demographic comparisons (Table 3) revealed significant differences: younger individuals (<30 years), males, higher-income earners, residents of the Southern region, and participants with Fitzpatrick skin types IV–VI had higher combined scores for sun behaviour and exposure (all *p* < 0.001). Specifically, younger adults exhibited riskier sun-related practices, and males had higher sun behaviour scores than females. Participants with previous skin cancer diagnosis showed notably higher sun exposure scores compared to those without such history (84.7 ± 45.1 vs. 47.8 ± 43.9; *p* < 0.001). It is important to note that higher SEBI scores denote greater cumulative sun exposure, and the elevated scores observed among participants with a previous diagnosis of skin cancer likely reflect their historical UV exposure rather than ongoing behaviour. Although such individuals may have modified their sun habits post-diagnosis, the SEBI captures both current and lifetime exposure, which may explain the persistence of high overall scores within this group.

**Figure 1:**
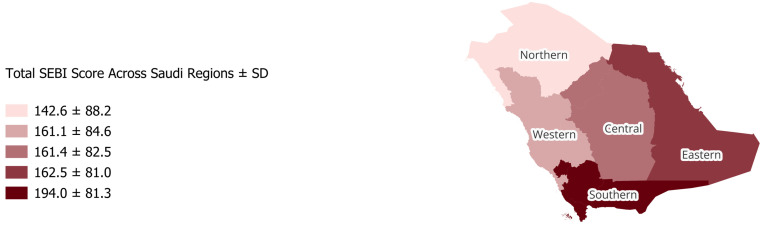
Geographic distribution of participants across Saudi Arabia by total SEBI score (±SD). The map illustrates regional variation in sun exposure behaviour across the Northern, Western, Central, Eastern, and Southern regions of the Kingdom. Major cities such as Riyadh, Jeddah, Dammam, Abha, and Tabuk are located within these regions. The vast Empty Quarter (Rub’ al Khali) occupies much of the southern desert area, while the country extends approximately from 16° N to 32° N latitude, encompassing diverse climatic zones that influence sun exposure patterns.

Additionally, individuals from higher-income and more educated backgrounds demonstrated better adherence to sun protection behaviours. The Southern region had the highest average combined score (194.0 ± 81.3), while the Northern region scored lowest overall (142.6 ± 88.2) (Figure 1). Analysis by Fitzpatrick skin type revealed a significant association with SEBI domain and total scores (*p* < 0.001). Participants with darker skin types generally demonstrated higher sun exposure scores. The mean total SEBI scores increased progressively from Type I to Type V, before slightly decreasing for Type VI. Specifically, participants with Type I skin had a mean total score of 151.7 ± 81.5, Type II 148.4 ± 80.4, Type III 169.6 ± 83.2, Type IV 197.0 ± 87.2, Type V 218.6 ± 76.0, and Type VI 196.9 ± 94.4. Higher SEBI domain and total scores reflect increased sun exposure or lower adherence to protective behaviours, whereas lower scores indicate more protective habits.

## 4. Discussion

This large cross-sectional study of 11,491 Saudi adults offers important insights into sun-exposure behaviours and protective practices across diverse regions. Findings revealed generally low adherence to sun-protection measures, despite high educational attainment. Sun exposure varied significantly by age, gender, income, skin type, and region. Younger adults, males, higher-income individuals, and residents of the Southern region reported significantly greater sun exposure. Notably, participants with darker Fitzpatrick skin types (IV–VI) also had higher exposure scores, reflecting the persistent misconception that darker skin provides sufficient protection against ultraviolet (UV) damage [20].

Compared to findings from Canada and other Western countries using the SEBI, the Saudi cohort reported markedly lower consistent sunscreen use (9.9%) and limited awareness of UVA/UVB protection (28.1%). Western populations benefit from stronger public health campaigns and different cultural perceptions of sun exposure, including tanning as a cosmetic practice [21,22]. In contrast, sun exposure in Saudi Arabia tends to be incidental, shaped by climate and lifestyle rather than aesthetic preferences. These findings differ substantially from SEBI-based studies conducted in Western populations. For instance, Jennings et al. [17] reported that 62% of Canadian respondents regularly used sunscreen, and 74% were aware that effective sunscreens should provide both UVA and UVB protection. In contrast, only 9.9% of participants in our Saudi cohort reported consistent sunscreen use, and 28.1% indicated awareness of UVA/UVB dual protection. Such differences likely reflect variations in public health messaging, cultural norms regarding sun exposure, and the influence of traditional clothing, which offers substantial physical protection from ultraviolet radiation in Saudi Arabia. These contextual factors may explain why sunscreen usage is less prevalent despite comparable or higher ambient UV levels. Furthermore, the present sample showed a higher proportion of females and younger adults, as well as a greater representation of university-educated participants. This demographic pattern is consistent with online survey-based studies conducted in Saudi Arabia, which often attract younger, more educated respondents, particularly females. Nonetheless, the wide geographic distribution across all regions supports the generalizability of findings regarding sun exposure behaviours within the Saudi population.

Although excessive UV exposure is a major risk factor for skin cancer, it is equally important to recognise that moderate sun exposure plays an essential physiological role—particularly in maintaining adequate vitamin D levels. Solar UVB radiation is the primary natural source of vitamin D, accounting for more than 80–90% of vitamin D synthesis in humans. This is especially relevant in Saudi Arabia, where a large body of research has consistently documented widespread vitamin D deficiency across different age groups, including healthy adults, children, pregnant women, and the elderly [23,24,25]. Despite abundant year-round sunlight, national and regional studies report vitamin D deficiency prevalence ranging from 60% to over 80%, often attributed to limited sun exposure, indoor lifestyles, and cultural clothing that reduces cutaneous UVB penetration. Comparable patterns have also been observed in neighbouring countries with similar climates and cultural contexts, such as the United Arab Emirates and Iran [26,27]. These findings highlight that the public-health conversation around sun exposure in Saudi Arabia must balance both the risks of excessive UV radiation and the health consequences of inadequate UVB-induced vitamin D synthesis.

Vitamin D deficiency has been linked to multiple adverse health outcomes, including reduced bone integrity, a higher likelihood of fractures, diminished immune function, and links to metabolic, cardiovascular, and autoimmune disorders. Notably, Alkalash et al. reported that vitamin D levels below ~20 ng/mL are associated with a 30–50% increased risk of colon, prostate, and breast cancers, along with higher mortality from these malignancies [24]. Several studies from the region—including those conducted in Saudi Arabia, Iran, and the UAE—emphasise the substantial public-health burden of vitamin D deficiency [24,26,27]. These trends reinforce the importance of maintaining safe yet adequate levels of sun exposure. In this context, sun-safety recommendations should encourage a balanced approach: promoting behaviours that minimise harmful UV exposure while still supporting sufficient UVB exposure for vitamin D synthesis. This includes brief, controlled sun exposure during lower-UV periods, combined with dietary and supplemental strategies when clinically indicated [25]. Integrating both risk-mitigation and health-promotion perspectives ensures a comprehensive approach consistent with the broader mission of healthcare, addressing dermatological risk without overlooking the essential physiological benefits of sunlight.

In addition to its systemic roles, emerging evidence suggests that the skin possesses local mechanisms for photoprotection. Gradual tanning through repeated non-burning UV exposure increases melanin content and can provide a modest natural protection equivalent to SPF 2–4. Furthermore, adequate vitamin D status may contribute to improved cellular responses to UV-induced damage, as the skin can locally convert 25-hydroxyvitamin D to its active form, which influences gene expression related to inflammation and DNA repair. While these physiological mechanisms may offer partial protection, they do not replace the need for evidence-based sun-safety practices.

While higher melanin levels in darker skin do provide greater natural protection against ultraviolet radiation, this photoprotective effect also reduces the efficiency of cutaneous vitamin D synthesis. Studies conducted in populations with darker pigmentation have shown differing patterns of UV-related risk, including lower susceptibility to UV-induced melanoma and a predominance of melanoma subtypes arising on non-sun-exposed sites, reflecting the protective influence of melanin [4,28]. At the same time, individuals with darker skin often underestimate their need for intentional sun protection and may be at increased risk of vitamin D deficiency due to reduced UVB penetration [29]. These patterns highlight the need for balanced public-health messaging that considers both melanin’s photoprotective role and the heightened risk of vitamin D insufficiency among darker skin types.

UVA and UVB radiation contribute differently to the development of skin cancers. UVB has a shorter wavelength and primarily induces direct DNA damage through cyclobutane pyrimidine dimers, playing a central role in the pathogenesis of non-melanoma skin cancers such as basal cell carcinoma and squamous cell carcinoma [4]. In contrast, UVA penetrates more deeply into the dermis and promotes oxidative stress and indirect DNA damage, mechanisms more strongly associated with melanoma development, particularly in individuals with lighter skin types [30,31]. Recognising these distinct biological pathways highlights the importance of broad-spectrum sunscreen use that provides protection against both UVA and UVB radiation.

An additional consideration is the variation in the spectral protection profiles of sunscreens commonly used in Saudi Arabia. Many preparations marketed locally provide strong UVB protection to prevent sunburn but offer limited UVA shielding, despite UVA being more strongly implicated in melanoma development due to its deeper dermal penetration and oxidative DNA damage pathways [4,31]. Limited public awareness of UVA protection has been reported in regional studies, with many users uncertain whether their sunscreen provides broad-spectrum coverage [31]. These factors underscore the importance of promoting sunscreens with adequate UVA and UVB filtration and improving public understanding of broad-spectrum labelling.

Socioeconomic and gender patterns also diverged from Western trends. While low-income groups in the West often face greater UV exposure due to outdoor work [31], in Saudi Arabia, higher-income participants reported greater exposure, likely from leisure activities [32]. Men exhibited higher sun exposure than women, echoing findings from local studies, although this differed from Western patterns, where women are more commonly intentional in seeking the sun [11].

Cultural and environmental factors play a critical role in shaping sun-protection behaviours. While traditional attire offers partial coverage, the face, neck, and hands remain vulnerable to UV exposure [33]. Misbeliefs about natural skin pigmentation providing adequate protection are common among darker-skinned individuals, similar to trends observed in other high-UV populations, such as Hispanic and Black communities in the U.S. [31].

These disparities underscore the need for culturally tailored public health initiatives. Campaigns should focus on high-risk groups—especially young adults, males, high-income earners, and those living in high-UV regions—emphasising the importance of sunscreen, understanding SPF, and dispelling myths around melanin and UV resistance [34]. Strategies proven effective in Western contexts, such as school-based interventions and targeted awareness campaigns, could be adapted locally [35]. Engaging trusted messengers—such as healthcare providers, religious figures, and social influencers—may improve message uptake.

While excessive ultraviolet (UV) radiation exposure remains a primary risk factor for skin malignancies, particularly melanoma, recent studies emphasise that the relationship between UV exposure and health outcomes is multifaceted. Lopes et al. [36] highlighted that UV exposure increases the risk of cutaneous melanoma even in individuals with darker skin tones, reinforcing the need for preventive awareness across all phototypes. Similarly, Cherrie and Cherrie [37] reported that occupational UV exposure contributes significantly to melanoma risk among outdoor workers, underscoring the importance of workplace sun protection strategies. Conversely, moderate UVB exposure is beneficial for maintaining adequate vitamin D levels, which supports bone and immune health. Raymond-Lezman et al. [38] noted that controlled sun exposure remains an important natural source of vitamin D, though excessive exposure negates its benefits by elevating cancer risk. Balancing these risks and benefits should inform future public health recommendations tailored to the Saudi population’s climatic and cultural context.

Addressing structural barriers is equally vital. Studies confirm that affordability and access have a strong influence on sunscreen use [9,39]. Policies to reduce cost and expand availability—especially in rural areas—could improve behaviour change. Digital tools, such as UV index apps and personalised alerts, offer promising low-cost strategies to reinforce protective habits [40].

In this context, mobile health technologies can serve as valuable tools for promoting sun-safe behaviours. The SunSmart Global UV App, jointly developed by the World Health Organization (WHO), World Meteorological Organization (WMO), and partner institutions, provides real-time UV index data and personalised sun protection recommendations. The app is freely available in multiple languages, including Arabic, which enhances accessibility for users in Saudi Arabia and other Arabic-speaking regions [41]. Incorporating such evidence-based digital tools into national public health campaigns could effectively improve awareness and behavioural adherence to sun protection guidelines.

This study demonstrates several strengths, including a large and geographically diverse sample from across Saudi Arabia, regionally representative sampling, and the use of the validated Arabic version of the SEBI (Ar-SEBI) to assess sun-exposure behaviours. Its analytical cross-sectional design is well-suited for capturing prevalence and associated factors at a single point in time, consistent with established observational research practices [42]. Despite these strengths, the study has limitations. Its cross-sectional nature limits causal interpretation, and reliance on self-reported data may introduce recall or social desirability bias. Moreover, while quota sampling ensured regional representation, it may have underrepresented groups such as outdoor labourers or rural residents with limited internet access. Nonetheless, the findings reveal critical disparities—particularly among young adults, males, and individuals with darker skin—and emphasise the need for culturally tailored health programmes. Public health initiatives should address misconceptions about sun protection, promote the correct use of sunscreen, and ensure access to affordable protective measures to reduce UV-related risks and encourage sustained sun-safe behaviours.

Future research should prioritise longitudinal and interventional designs to assess the impact of educational and behavioural strategies on real-world sun-protection practices [43]. Further investigation into psychosocial determinants—such as perceived invulnerability, cultural norms regarding tanning, and trust in health messaging—would deepen understanding [44]. The integration of digital tools into behaviour change frameworks may also provide scalable solutions for public health efforts [40].

## 5. Conclusions

This study highlights important gaps in sun-protection behaviours among the Saudi Arabian population. Despite a general familiarity with sun-safety concepts, practical adherence—such as regular sunscreen use, seeking shade, and wearing protective clothing—remains limited. These behaviours were notably lacking among males, younger adults, and residents of southern regions, where UV exposure is typically highest. Misconceptions about the protective role of melanin and limited awareness of the need for daily sun protection contribute to the underuse of preventive measures.

The findings may inform context-specific public health strategies and offer insights for adapting global sun-safety recommendations to better align with local climatic conditions and cultural norms.

## Figures and Tables

**Table 1:** Demographic Characteristics and Skin Type of the Study Population.

			Total Number (%)
Age (years) *	<30	29.6 ± 10.9	6508 (57%)
	>30		4936 (43%)
Gender	Male		3318 (29%)
	Female		8173 (71%)
Level of education	High school and below		2908 (25%)
	Bachelor’s degree		8005 (70%)
	Postgraduate degree		578 (5%)
Monthly income/allowance	Less than 5000 SAR		6965 (61%)
	From 5000 to 15,000 SAR		2998 (26%)
	From 15,000 to 30,000 SAR		1194 (10%)
	More than 30,000 SAR		334 (3%)
Residence **	Central region		3076 (27%)
	Northern region		1565 (14%)
	Southern region		2147 (19%)
	Eastern region		2569 (22%)
	Western region		2115 (18%)
Fitzpatrick skin type	Type I		1187 (10%)
	Type II		3931 (34%)
	Type III		4653 (41%)
	Type IV		1334 (12%)
	Type V		300 (3%)
	Type VI		86 (1%)
Previous Skin Cancer	Yes		32 (0%)
	No		11,459 (100%)
Current Immunosuppressive Therapy	Yes		411 (4%)
	No		11,080 (96%)

* Missing 47 responses. ** Missing 19 responses.

**Table 2:** Sun Exposure and Protective Behaviours Among Participants.

Item		Number (%)	Mean (SD)
SEBI 1: When you go outside on a warm, sunny summer day (May-September) for more than one hour, how often do you use sunscreen or sun block?	Do not stay out > an hour	2781 (24%)	27 ± 20
	Always	1143 (10%)	
	Nearly always	1226 (11%)	
	Sometimes	1715 (15%)	
	Seldom	1456 (13%)	
	Do not know	3170 (28%)	
SEBI 2: What is the Sun Protection Factor or SPF of the sunscreen that you use most often?	SPF 15 or higher	3134 (27%)	4 ± 2
	<SPF 15/do not know	8357 (73%)	
SEBI 3: Does your sunscreen have both UVA and UVB Protection?	Yes	3234 (28%)	4 ± 2
	No/do not know	8257 (72%)	
SEBI 4: When you go outside on a sunny summer day for more than an hour, how often do you stay in the shade?	Do not stay out > an hour	2955 (26%)	17 ± 13
	Always	1929 (17%)	
	Nearly always	3238 (28%)	
	Sometimes	2485 (22%)	
	Seldom	580 (5%)	
	Do not know	304 (3%)	
SEBI 5: When you go outside on a sunny summer day for more than an hour, how often do you wear a wide-brimmed hat or any other hat that shades your face, ears, and neck from the sun?	Do not stay out > an hour	1600 (14%)	19 ± 16
	Always	4960 (43%)	
	Nearly always	1328 (12%)	
	Sometimes	1425 (12%)	
	Seldom	731 (6%)	
	Do not know	1447 (13%)	
SEBI 6: When you go outside on a sunny summer day for more than an hour, how often do you wear long-sleeved shirts?	Do not stay out > an hour	1182 (10%)	18 ± 15
	Always	6449 (56%)	
	Nearly always	975 (9%)	
	Sometimes	1015 (9%)	
	Seldom	517 (5%)	
	Do not know	1353 (12%)	
SEBI 7: Have you been tan in the past 12 months?	No	8067 (70%)	12 ± 18
	Yes	3424 (30%)	
SEBI 8: During the months of May-September, how many days per week did you do outdoor activities, i.e., gardening, golf, beach activities, and outdoor sports?	0	5189 (45%)	16 ± 20
	1	1327 (12%)	
	2	1639 (14%)	
	3	1398 (12%)	
	4	807 (7%)	
	5	458 (4%)	
	6	168 (2%)	
	7	505 (4%)	
SEBI 9: During the months of October-April, how many days per week did you do outdoor activities (i.e., gardening, golf, beach activities, outdoor sports)?	0	4571 (40%)	20 ± 22
	1	1415 (12%)	
	2	1453 (13%)	
	3	1409 (12%)	
	4	972 (9%)	
	5	692 (6%)	
	6	293 (3%)	
	7	686 (6%)	
SEBI 10: Total number of sunburns (sunburns is defined as skin redness or pain which lasts at least two days after sun exposure) you have had in your life:	0 sunburns	8018 (70%)	4 ± 6
	1–10 sunburns	3061 (27%)	
	11–20 sunburns	266 (2%)	
	More than 20 sunburns	146 (1%)	
SEBI 11: Total number of blistering sunburns you have had in your life:	0 blistering sunburn	9460 (82%)	22 ± 10
	1–3 blistering sunburn	1506 (13%)	
	4–10 blistering sunburn	173 (2%)	
	>10 blistering sunburn	352 (3%)	
SEBI 12: Total number of times you have used a tanning bed/booth/sunlamp in your life:	0 times	10,684 (93%)	1 ± 2
	1–5 times	492 (4%)	
	6–12 times	223 (2%)	
	More than 20 times	92 (1%)	
SEBI 13: Have you lived in an area with a different climate from MENA region (i.e., Riyadh, eastern/western side, Caribbean, North Africa, and Southeast Asia) for 6 months or longer? *	No	3082 (27%)	1 ± 0.4
	Yes	8409 (73%)	
If yes to SEBI 13: How long?	-	2237 (20%)	6 ± 4
	Less than 5 years	1783 (16%)	
	6–10 years	888 (8%)	
	11–20 years	2013 (18%)	
	More than 20 years	4570 (40%)	
SEBI 14: Compared to other people of your age and sex, how would you rate your lifetime sun exposure?	A lot less exposure than the average person	1629 (14%)	8 ± 9
	Less exposure than the average person	2534 (22%)	
	The same exposure as the average person	5093 (44%)	
	More exposure than the average person	1165 (10%)	
	A lot more exposure than the average person	1070 (9%)	
SEBI 15: Compared to other people your age and sex, how would you rate your lifetime sun exposure from outdoor recreation? (examples: golf, gardening, outdoor sports, beach activities, etc	A lot less exposure than the average person	2758 (24%)	
	Less exposure than the average person	2844 (25%)	
	The same exposure as the average person	3741 (33%)	6 ± 10
	More exposure than the average person	1191 (10%)	
	A lot more exposure than the average person	957 (8%)	
SEBI 16: Compared to other people your age and sex, how would you rate your lifetime sun exposure from your job? (examples of high exposure jobs: fishermen, construction workers, etc.)	A lot less exposure than the average person	4143 (36%)	
	Less exposure than the average person	2224 (19%)	
	The same exposure as the average person	3321 (29%)	5 ± 10
	More exposure than the average person	966 (8%)	
	A lot more exposure than the average person	837 (7%)	

* Note: Item excluded from analysis due to multiple responses.

**Table 3:** Comparison of Total Sun Behaviour and Exposure Scores by Demographic Factors.

Variable		(A) Current Sun Exposure	*p*-Value	(B) Current Sun Behaviour	*p*-Value	(C) Prior Sun Exposure	*p*-Value	(D) Total Score of Overall	*p*-Value
Age (years)	<30	49 ± 44	*p* < 0.001	89 ± 47	*p* < 0.001	31 ± 29	*p* < 0.001	169 ± 86	*p* < 0.001
	>30	46 ± 44		86 ± 45		28 ± 25		160 ± 82	
Gender	Male	63 ± 44	*p* < 0.001	122 ± 46	*p* < 0.001	40 ± 29	*p* < 0.001	225 ± 81	*p* < 0.001
	Female	42 ± 42		74 ± 39		25 ± 26		141 ± 73	
Level of education	High school and below	47 ± 46	*p* < 0.001	88 ± 46	*p* = 0.19	29 ± 29	*p* < 0.001	164 ± 85	*p* < 0.001
	Bachelor degree	48 ± 43		87 ± 46		30 ± 26		164 ± 84	
	Postgraduate degree	57 ± 48		91 ± 50		27 ± 30		185 ± 93	
Monthly income	<5000 SAR	45 ± 44	*p* < 0.001	85 ± 46	*p* < 0.001	28 ± 28	*p* < 0.001	158 ± 84	*p* < 0.001
	5000–15,000 SAR	49 ± 43		89 ± 44		30 ± 26		168 ± 82	
	15,000–30,000 SAR	57 ± 45		96 ± 48		36 ± 28		189 ± 85	
	>30,000 SAR	62 ± 49		98 ± 54		41 ± 32		201 ± 95	
Residence region	Central	47 ± 44	*p* < 0.001	85 ± 47	*p* < 0.001	30 ± 27	*p* < 0.001	161 ± 83	*p* < 0.001
	Northern	44 ± 45		74 ± 44		24 ± 29		143 ± 88	
	Southern	56 ± 41		105 ± 45		33 ± 28		194 ± 81	
	Eastern	46 ± 44		86 ± 45		30 ± 27		163 ± 81	
	Western	46 ± 45		87 ± 45		29 ± 27		162 ± 85	
Fitzpatrick skin type	Type I	45 ± 44	*p* < 0.001	78 ± 45	*p* < 0.001	28 ± 27	*p* < 0.001	152 ± 82	*p* < 0.001
	Type II	43 ± 43		79 ± 43		27 ± 27		148 ± 80	
	Type III	49 ± 44		91 ± 46		30 ± 27		170 ± 83	
	Type IV	57 ± 45		104 ± 48		36 ± 29		197 ± 87	
	Type V	65 ± 40		117 ± 43		27 ± 28		219 ± 76	
	Type VI	62 ± 51		100 ± 49		36 ± 31		197 ± 94	
skin cancer	Yes	85 ± 45	*p* < 0.001	86 ± 31	*p* = 0.93	77 ± 34	*p* < 0.001	248 ± 84	*p* < 0.001
	No	48 ± 44		88 ± 46		29 ± 27		165 ± 84	
immunosuppressant	Yes	50 ± 48	*p* = 0.65	81 ± 46	*p* = 0.003	37 ± 33	*p* < 0.001	168 ± 88	*p* = 0.57
	No	48 ± 44		88 ± 46		29 ± 27		165 ± 84	

## Data Availability

Data used in this study are available upon reasonable request from the corresponding author.
